# Tolerance and the effect of high doses of wheat bran extract, containing arabinoxylan–oligosaccharides, and oligofructose on faecal output: a double-blind, randomised, placebo-controlled, cross-over trial

**DOI:** 10.1017/jns.2014.52

**Published:** 2014-10-20

**Authors:** Isabelle E. J. A. François, Olivier Lescroart, Wim S. Veraverbeke, Karen Windey, Kristin Verbeke, Willem F. Broekaert

**Affiliations:** 1 FUGEIA NV, Kluizenbosstraat 26, B-1700 Dilbeek, Belgium; 2 Translational Research for Gastrointestinal Disorders (Targid) and Leuven Food Science and Nutrition Centre (LFoRCe), University Hospitals UZ Leuven, Herestraat 49, O & N1, Box 701, B-3000 Leuven, Belgium

**Keywords:** Arabinoxylan–oligosaccharides, Wheat bran extract, Gastrointestinal tolerance, Oligofructose, Prebiotics, AE, adverse event, AXOS, arabinoxylan–oligosaccharides, EE, efficacy evaluable, GI, gastrointestinal, PP, per protocol, WBE, wheat bran extract

## Abstract

Wheat bran extract (WBE) is a food-grade soluble fibre preparation that is highly enriched in arabinoxylan–oligosaccharides. In this placebo-controlled cross-over human intervention trial, tolerance to WBE as well as the effects of WBE on faecal parameters, including faecal output and bowel habits, were studied. After a 2-week run-in period, twenty healthy volunteers consumed WBE (15 g/d in the first week, 30 g/d in the second week), oligofructose (15 g/d in the first week, 30 g/d in the second week) and placebo (for 2 weeks) in a random order, with 2-week washout periods between each treatment period. Subjects collected a 72 h stool sample for analysis of faecal output, stool pH and stool moisture concentration. Additionally, the volunteers completed questionnaires scoring occurrence frequency and distress severity of eighteen gastrointestinal (GI) symptoms. An overall GI symptom measure was calculated to analyse the overall effect of WBE and oligofructose on GI symptoms. Intake of both 30 g/d WBE and 30 g/d oligofructose lowered stool pH, indicative of increased colonic fermentation, and increased stool moisture concentration as compared with placebo intake. Intake of 30 g/d oligofructose increased the overall GI symptom measure by 1·9-fold as compared with placebo intake. Intake of WBE at doses up to 30 g/d did not affect the overall GI symptom measure. WBE exerts beneficial effects on stool characteristics and is well tolerated at up to 30 g/d. Oligofructose exerts comparable beneficial effects on stool characteristics. However, intake of 30 g/d oligofructose appears to cause GI discomfort to some extent.

Wheat bran extract (WBE) is a food-grade preparation that is highly enriched in arabinoxylan–oligosaccharides (AXOS) and that is produced by enzymic extraction from wheat bran. The AXOS in WBE consist of a backbone of β-1,4-linked d-xylopyranosyl residues, some of which are mono- or di-substituted at the C(*O*)2 and/or C(*O*)3 position with α-l-arabinofuranosyl residues^(^^1^^–^^4^^)^.

AXOS are non-digestible, fermentable prebiotic oligosaccharides, of which the bifidogenic activity has been demonstrated *in vitro*^(^^5^^)^, in animals^(^^6^^,^^7^^)^, in healthy adults^(^^8^^–^^10^^)^ and in healthy children^(^^11^^)^. Next to bifidogenic activity, AXOS have been shown to increase the faecal levels of acetic acid, propionic acid and butyric acid^(^^10^^)^, in turn leading to acidification of the colonic lumen. In addition, AXOS appear to reduce colonic protein fermentation in human subjects^(^^8^^,^^10^^,^^12^^)^.

For comparative purposes, WBE was in the present study analysed alongside oligofructose. Oligofructose is composed of β-1,2-linked fructose with a terminal α-1,2-glucose. It is derived by partial hydrolysis from inulin, a polysaccharide that occurs naturally in vegetables such as chicory, onion and artichoke and that is industrially mainly extracted from chicory roots. Together with inulin, oligofructose is the most extensively studied and most widely used prebiotic compound, and its beneficial effects on humans have been thoroughly documented^(^^13^^–^^18^^)^.

Some studies have indicated that prebiotics can be mildly laxative. In two out of six published clinical trials with the prebiotic compounds oligofructose, inulin, galacto-oligosaccharides and *trans*-galacto-oligosaccharides, a significant increase in faecal output was observed (for a review, see Macfarlane *et al.*^(^^19^^)^). Xylo-oligosaccharides, a prebiotic compound with structural similarity to AXOS, has been proven to alleviate constipation in Japanese pregnant women^(^^20^^)^. Till now, the effect of WBE on faecal output has been investigated in only one study in which healthy volunteers ingested 14 g/d WBE for 3 weeks^(^^8^^)^. In this study WBE had no effect on faecal output, which could possibly be attributed to the relatively low dosage of WBE used in this study.

The aim of the present study was to analyse the effect of a high WBE dose, in comparison with the same dose of oligofructose, on faecal output in healthy volunteers. In addition, the effect of intake of WBE and oligofructose on stool pH, stool consistency, defecation frequency and stool moisture concentration was analysed. Since intake of high doses of prebiotics can potentially cause gastrointestinal (GI) problems, the tolerance of the volunteers to the high doses of WBE and oligofructose was also assessed in the present study.

## Experimental methods

### Composition of wheat bran extract

Wheat bran extract (WBE; Brana Vita^®^ 200) was produced from wheat bran by Fugeia NV, using a procedure based on that previously described^(^^1^^)^. WBE was analysed for the content of arabinoxylans–oligosaccharides (AXOS), the AXOS average degree of polymerisation, its arabinose:xylose ratio, its content of bound glucuronic acid, bound ferulic acid, glucose as part of poly/oligosaccharides, mannose as part of poly/oligosaccharides, galactose as part of poly/oligosaccharides, free monosaccharides, moisture, protein, ash and lipid by analytical procedures outlined previously^(^^10^^,^^21^^)^.

Table 1 shows the composition of the WBE preparation used in the present study. It consisted of 80·9 % AXOS (on a DM basis), had an average degree of polymerisation of 5 and an arabinose:xylose ratio of 0·20.

**Table 1.** Characterisation of the wheat bran extract preparation

Constituents	Composition (% DM)
AXOS	80·9
Of which xylo-oligosaccharides (XOS_DP3–9_)	34·1
Of which xylobiose (XOS_DP2_)	18·6
Average DP of AXOS	5
Arabinose:xylose ratio of AXOS	0·20
Glucuronic acid bound to AXOS	1·1
Ferulic acid bound to AXOS	1·4
Glucose as part of poly/oligosaccharides	13·6
Galactose as part of poly/oligosaccharides	0·5
Mannose as part of poly/oligosaccharides	0·3
Total free monosaccharides	1·4
Protein	0·5
Total lipids	< 0·5
Ash	0·1
DM	98·7

AXOS, arabinoxylans–oligosaccharides; XOS, xylo-oligosaccharides; DP, degree of polymerisation.

Frutalose^®^ L92 (Sensus) is an oligofructose preparation produced by partial hydrolysis of chicory inulin. Oligofructose from chicory inulin is a polydisperse mixture of linear β(2-1)-linked fructose polymers partly ended by a glucose molecule. The degree of polymerisation varies between 2 and 10. The composition of Frutalose^®^ L92 is given in Table 2.

**Table 2.** Composition of Frutalose^®^ L92 (oligofructose preparation)

Constituents	Composition (g/100 g Frutalose^®^ L92 syrup)
Carbohydrates	75
Non-digestible (oligofructose)	69
Digestible (sugars)	6
Proteins	0
Lipids	0
Moisture	25

### Subjects

Based on the datasets of earlier human intervention trials with WBE and WBE-like material^(^^8^^,^^22^^)^, an evaluable sample size of fifteen was expected to provide 80 % power (two-sided, α = 0·05) for detecting a statistically significant difference in faecal output (the primary outcome variable of the present study) between 30 g/d WBE treatment and placebo treatment. Based on this power analysis, it was decided to recruit approximately eighteen individuals for the present trial. A total of twenty individuals (ten women and ten men; mean age 46·9 (sd 15·9) years; mean BMI 24·4 (sd 3·4) kg/m^2^; all of Caucasian ethnicity) participated in the study. Exclusion criteria were a low-energy diet or other extreme dietary habits in the 6 weeks before the start of the clinical trial, intake of antibiotics in the 3 months before the start of the clinical trial, intake of medication or dietary supplements influencing GI tract processes in the 2 weeks before the start of the clinical trial, history of abdominal surgery (with the exception of appendectomy), serious illness (defined as being unable to work for more than 2 weeks) in the 3 months before the start of the clinical trial, complete anaesthesia in the month before the start of the clinical trial, history of chronic GI conditions such as inflammatory bowel disease and irritable bowel syndrome, allergy to wheat products, coeliac disease, alcohol abuse or smoking of more than five cigarettes per d. Female volunteers were excluded if pregnant or lactating. During the study, the subjects ate their usual diet, but were asked to have a regular eating pattern (three meals per d). The intake of food products containing probiotics and/or prebiotics was prohibited. At the time of inclusion, all subjects were informed about pro- and prebiotics and the food products containing them. The subjects were asked to read food labels carefully to check for absence of pro- and/or prebiotics. The present study was conducted according to the guidelines laid down in the Declaration of Helsinki and all procedures involving human subjects were approved by the Ethics Committee of the University Hospitals UZ Leuven, Belgium (approval no. ML6521). Written informed consent was obtained from all subjects. Compliance was assessed by inquiry and by counting the returned containers, in which the study product was supplied to the volunteers, at the end of each treatment period. Non-compliance was defined as not taking 100 % of the study products during at least 11 of the 14 d of a treatment period and/or not taking 80 % of the study products during the 2 d before stool collection and during the 3 d of the stool collection.

### Study design

**Fig. 1.**
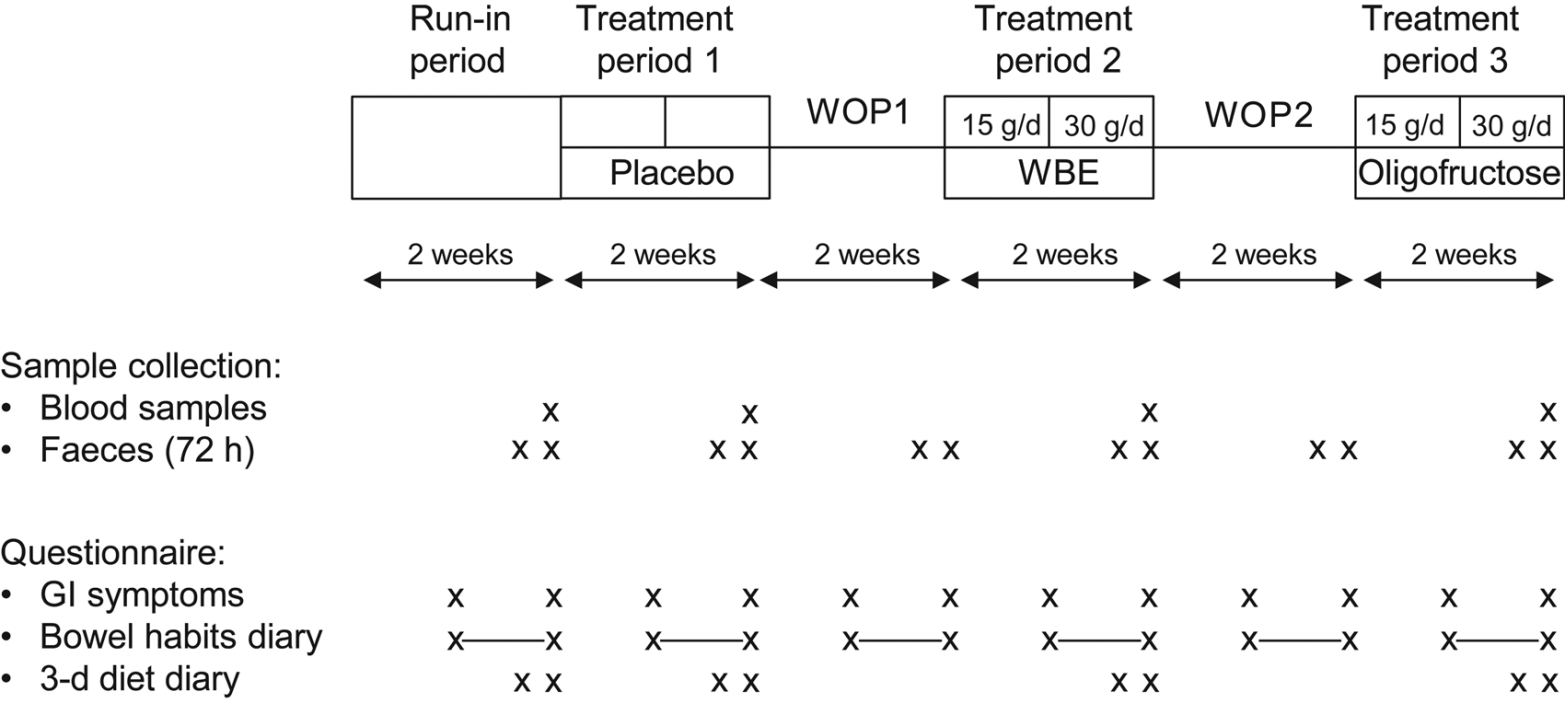
Schematic representation of the study design. The study started with a 2-week run-in period, followed by three 2-week treatment periods in which the following study products (not necessarily in the described order) were taken by the volunteers: (i) wheat bran extract (WBE) at a dose of 15 g/d (first week of WBE treatment period) and 30 g/d (second week of WBE treatment period); (ii) oligofructose at a dose of 15 g/d (first week of oligofructose treatment period) and 30 g/d (second week of oligofructose treatment period); and (iii) placebo. The treatment periods were separated by 2-week washout periods (WOP). Blood and faecal samples were collected at the indicated time points. The subjects completed weekly a questionnaire assessing the occurrence frequency and distress severity of eighteen gastrointestinal (GI) symptoms. Additionally, subjects recorded in the bowel habits diary the number of bowel movements and stool consistency during the second week of the run-in period, and each of the treatment periods and washout periods.

Fig. 1 presents a schematic overview of the randomised, placebo-controlled, double-blind, cross-over study. The study started with a 2-week run-in period, followed by three 2-week treatment periods with 2-week washout periods in between two consecutive treatment periods. Each subject underwent three treatment periods. During the 2-week treatment periods, the following treatments were applied, yet not necessarily in this order: placebo treatment; treatment with WBE (first week: 15 g/d; second week: 30 g/d); and treatment with oligofructose (first week: 15 g/d; second week: 30 g/d). Clinic visits took place at the end of the run-in period, at the end of each washout period and at the end of each treatment period. WBE, oligofructose and placebo were administered as non-carbonated soft drinks, of which the volunteer had to drink three times daily 170 ml, once after breakfast, once after lunch and once after dinner. Consumption of the soft drinks took place at the volunteers' homes. The soft drinks contained sucrose, colorant, flavour, citric acid and clouding agent (PE5273; Metarom). The placebo soft drink had the same composition as the WBE- and oligofructose-containing soft drinks, except for the omission of WBE and oligofructose. Volunteers were randomly assigned to one of six randomisation groups, each randomisation group differing in the sequence by which the three types of drinks were to be consumed. A list of unique three-digit numbers (volunteer numbers) was generated in a random way. Each volunteer number was linked with the number of a randomisation group. At the screening visit, an eligible subject who gave informed consent was randomly assigned to a volunteer number.

### Collection of faecal and blood samples

From the morning of day 12 till the morning of day 15 of the run-in period and of each of the two washout and three treatment periods, subjects collected all bowel movements (= 72 h collection). The faecal samples were frozen after defecation, and kept frozen until delivery at the clinic. On the morning of day 15 of the run-in period and of each of the three treatment periods, fasting serum and plasma samples were collected from the subjects. EDTA, lithium heparin and fluoride/oxalate were used as anti-coagulants. Immediately after blood collection, plasma and serum were transferred to the central laboratory facility of the University Hospitals UZ Leuven for analysis of the haematological and clinical chemistry parameters listed in Table 3.

**Table 3.** Haematological and clinical blood chemistry parameters following intake of placebo, wheat bran extract (WBE) at 30 g/d or oligofructose at 30 g/d (Mean values and standard deviations)

	Placebo treatment period		30 g/d WBE treatment period		30 g/d oligofructose treatment period		P*			
	Mean	sd	Mean	sd	Mean	sd	*P1*	*P2*	*P3*	P4
Haematological parameters										
Platelets (10^9^/l)	245·7	57·5	241·4	50·1	245·7	61·4	0·608	0·762	0·959	0·591
Eosinophils (%)	2·1	1·6	2·9	1·6	2·5	1·8	0·392	0·363	0·919	0·599
Hb (g/l)	138·5	11·0	136·5	13·0	136·9	10·9	0·474	0·434	0·745	0·871
Lymphocytes (%)	31·9	9·6	34·2	9·9	33·8	9·1	0·348	0·352	0·476	0·975
MCH (pg)	29·5	1·6	29·5	1·8	29·6	1·7	0·738	0·770	1·000	0·781
MCHC (g/l)	334·7	8·7	335·8	10·4	335·0	8·9	0·953	0·961	0·961	1·000
MCV (fl)	88·1	4·6	87·9	4·7	88·4	4·8	0·156	0·120	0·774	0·402
Monocytes (%)	8·8	1·8	9·6	2·7	10·0	3·3	0·479	0·797	0·436	0·827
MPV (fl)	11·2	0·7	11·3	0·7	11·2	0·8	0·168	0·177	0·982	0·248
Erythrocyte count (10^9^/l)	4·7	0·5	4·6	0·5	4·6	0·4	0·435	0·413	0·629	0·934
Erythrocyte distribution width (%)	13·3	0·5	13·5	0·6	13·3	0·6	0·157	0·451	0·718	0·117
Leucocyte count (10^9^/l)	5·9	1·8	5·5	1·5	5·6	1·7	0·385	0·417	0·470	0·996
Clinical chemistry parameters										
Glucose (mg/l)	894·7	111·9	860·0	132·9	854·2	144·2	0·546	0·654	0·556	0·987
Insulin (pmol/l)	56·3	34·0	53·5	36·1	51·4	20·1	0·932	0·933	0·997	0·956
Ca (mg/l)	90·3	3·6	89·3	2·4	89·2	2·7	0·563	0·602	0·630	0·999
Cl (mm)	104·4	1·9	104·4	2·7	104·7	2·4	0·900	0·975	0·967	0·891
Fe (µg/l)	1013·7	415·5	1127·0	523·3	941·1	362·8	0·469	0·425	0·777	0·836
K (mm)	4·5	0·3	4·5	0·3	4·5	0·3	0·921	0·996	0·921	0·951
Mg (mg/l)	20·0	1·6	19·8	1·6	20·1	1·5	0·394	0·348	0·813	0·720
Na (mm)	142·0	2·0	141·8	1·6	141·9	1·7	0·973	0·974	0·999	0·983
Phosphate (mg/l)	35·1	5·5	34·4	4·9	34·7	6·3	0·874	0·909	0·881	0·998
Bicarbonate (mm)	25·8	1·7	26·1	2·1	25·8	2·4	0·660	0·682	0·996	0·733
Cholesterol (mg/l)	1873·7	404·5	1982·5	443·4	1972·6	423·6	0·158	0·445	0·119	0·728
HDL-cholesterol (mg/l)	640·5	187·9	645·5	194·1	653·2	171·4	0·448	0·515	0·999	0·496
LDL-cholesterol (mg/l)	1024·0	297·7	1108·0	314·2	1131·9	304·7	0·093	0·595	0·058	0·388
TAG (mg/l)	849·4	400·8	920·5	604·1	762·8	389·4	0·657	0·989	0·665	0·750
γ-Glutamyltransferase (U/l)	42·3	95·6	74·4	221·4	34·4	46·4	0·886	0·990	0·936	0·881
Lactate dehydrogenase (U/l)	330·2	42·7	320·8	50·8	323·1	51·5	0·392	0·353	0·656	0·871
Phosphatase (U/l)	174·9	44·5	171·8	41·2	182·4	48·6	0·501	0·986	0·512	0·614
Alanine aminotransferase (U/l)	21·6	10·9	28·3	27·0	25·7	16·5	0·882	0·975	0·956	0·871
Amylase (U/l)	64·1	23·9	64·5	17·8	67·6	26·2	0·288	0·960	0·208	0·453
Aspartate aminotransferase (U/l)	25·9	13·7	33·9	39·9	26·9	13·6	0·344	0·295	0·699	0·773
Creatine kinase (U/l)	103·7	54·9	109·6	82·5	97·4	49·2	0·267	0·280	0·368	0·983
Lipase (U/l)	33·9	7·5	34·3	9·4	35·3	10·9	0·976	0·976	0·998	0·986
Folate (µg/l)	9·0	2·4	9·1	3·0	9·1	2·3	0·959	0·969	0·963	1·000
Vitamin A (µg/l)	515·1	120·1	521·7	155·8	512·0	141·5	0·842	0·894	0·994	0·845
Albumin (g/l)	48·8	3·0	48·2	2·6	48·2	2·9	0·391	0·347	0·832	0·696
Bilirubin (mg/l)	5·7	1·9	6·1	2·5	5·3	2·1	0·938	0·997	0·963	0·938
Total protein (g/l)	71·6	3·0	70·8	3·0	70·8	3·4	0·462	0·418	0·803	0·803
Uric acid (mg/l)	48·3	10·8	49·8	14·1	49·1	12·8	0·919	0·912	0·967	0·985
Creatinine (mg/l)	8·2	1·6	8·0	1·6	8·0	1·7	0·943	0·942	0·967	0·996
Urea (mg/l)	302·1	85·5	306·5	84·7	301·6	84·4	0·814	0·976	0·907	0·803
Vitamin E (mg/l)	10·1	3·5	10·8	4·6	10·1	3·0	0·463	0·830	0·419	0·777
Vitamin B_12_ (ng/l)	434·5	215·0	422·1	179·3	451·9	230·1	0·361	0·521	0·954	0·351

MCH, mean corpuscular Hb; MCHC, mean corpuscular Hb concentration; MCV, mean corpuscular volume; MPV, mean platelet volume.

* *P1* is the *P* value of the conditional *F* test for overall significant treatment-related effects; *P2*, *P3* and *P4* are the *P* values for the comparison between 30 g/d WBE and placebo, 30 g/d oligofructose and placebo, and 30 g/d WBE and 30 g/d oligofructose, respectively.

### Recording of adverse events

Subjects were asked to record whether they had suffered from a medical condition (differing from baseline recordings), had to take new medication or had to stop taking previously reported medication. Additionally, at each clinic visit, the subjects were asked these questions. This information was recorded in the appropriate section of the case report form.

### Biochemical analyses of faecal and blood samples

To determine the faecal output (g stool/d), the total wet weight of the 72 h stool collection was determined. For each 72 h collection, the last collected faecal sample was used to determine faecal pH. To this end, one part of faeces (approximately 1 g) was homogenised by mixing with nine parts on a weight basis of demineralised water^(^^23^^)^. The pH was measured immediately upon homogenisation. The remaining faecal fractions of each 72 h collection were mixed and homogenised before determination of faecal moisture, which was determined as the ratio of the weight loss of the faecal sample after lyophilisation to the weight of the faecal sample before lyophilisation.

Haematological parameters, clinical blood chemistry, blood lipids, blood vitamins and blood minerals were analysed using standard laboratory techniques for blood analysis.

### Recording of gastrointestinal symptoms and calculation of overall gastrointestinal symptom measure

A questionnaire scoring the following eighteen GI symptoms was used: diarrhoea; constipation; painful bowel movement; blood in stool; abdominal pain; bloating; abdominal cramps; abdominal stretching; borborygmi; flatulence; burping; acid regurgitation; retrosternal burning; nausea; vomiting; indigestion; difficulty with swallowing; and hoarseness/sore throat. Occurrence frequency of the GI symptoms was scored on a five-step scale ranging from never (0), occasionally (1), frequently (2), nearly always (3) to always (4). Distress severity of the GI symptoms was graded on a five-step scale ranging from no (0), minimal (1), mild (2), moderate (3) to severe (4) distress. This GI symptom questionnaire was used in a previous study^(^^10^^)^. Subjects were asked to grade these GI symptoms weekly during the trial. In order to compare the overall severity of the GI symptoms across the treatments, an overall average GI symptom measure was calculated by averaging over the appropriate treatment period the occurrence frequency and distress severity scores of all of the eighteen symptoms.

### Recording of stool parameters

During the last week of the run-in period and during the last week of each treatment and washout period, defecation frequency as well as stool consistency (according to the Bristol Stool Form Scale^(^^24^^)^) were recorded daily using appropriate questionnaires. Average defecation frequency was calculated as the number of stools divided by the numbers of days of diary recording. Average stool consistency was calculated as the sum of Bristol Stool Form Scales divided by the number of stools. The composite parameter of defecation frequency and stool consistency (also called the Bristol Composite Measure) was calculated as the sum of Bristol Stool Form Scales divided by the number of days of diary recording^(^^25^^)^.

### Dietary composition

Subjects were asked to record all food and beverage intake of days 12 to 14 of the run-in period and of each treatment period. These data were used to calculate the average daily energy intake (kJ), the average percentage of energy from carbohydrates, the average percentage of energy from lipids, the average percentage of energy from proteins and the fibre content (g). These calculations were made using Vodisys Medical Software (Becel). During the 30 g/d WBE treatment period an additional 630·5 kJ/d, 23·7 g/d carbohydrates and 27·5 g/d fibre were added to the reported data; during the 30 g/d oligofructose treatment period, an additional 515·8 kJ/d, 17·4 g/d carbohydrates and 26·8 g/d fibre were added to the reported data; during the placebo treatment period, an additional 376·8 kJ/d, 22·5 g/d carbohydrates and 0 g/d fibre were added to the reported data. The energy value of WBE and oligofructose was derived from their content in fibre (8·5 kJ/g), carbohydrates except non-digestible disaccharides (17 kJ/g), non-digestible disaccharides (8·5 kJ/g) and protein (17 kJ/g).

### Statistical analysis of efficacy variables

In order to test for differences at baseline, the treatment sequence groups were compared with respect to age, sex, BMI and faecal output. Comparison of the groups was based on a one-way ANOVA and Fisher's exact test in the case of sex.

All tests of significance were performed at α = 0·05 (two-sided), unless otherwise stated. Assumptions of normality of residuals were investigated for each variable using the Shapiro–Wilk test^(^^26^^)^. When data were normally distributed, linear mixed models were applied to the raw data as such. When data were not normally distributed, values were rank-transformed and the linear mixed model was performed on the rank-transformed data^(^^27^^)^. Ties occurring during the rank transformation were replaced with their average rank. The data to estimate the fixed effect parameter for the run-in of the response remained unranked.

Evaluations of the effects of treatment on the efficacy variables were completed on an efficacy evaluable (EE) population, defined as all randomised subjects who received at least one serving of study product and provided at least one post-randomisation outcome data point during each treatment period. Volunteers who had to use antibiotics were excluded from the EE population. Evaluations of the treatment effects on the efficacy variables were also completed on a per protocol (PP) population, a subset of the EE subjects who completed the study, were compliant (as defined above), who did not take prohibited medication or products and had no major protocol violations.

### Continuous efficacy measurements from the faecal sample, the blood sample, the 3 d diet diary, the bowel habits diary, and the overall gastrointestinal symptom measure

Statistical analysis of continuous efficacy variables was performed using linear mixed models, which has the advantage over more traditional approaches, such as repeated-measures ANOVA, that it takes into account in a unified framework the fact that repeated measures on the same subject (within-subject measures, as typically performed in cross-over studies) are correlated, and that it is less vulnerable to impact from missing values. Treatment effects as well as treatment by treatment sequence interaction effects were tested with linear mixed models using conditional *F* and *t* tests^(^^28^^)^ (significance at α = 0·1). The single-step Tukey *post hoc* multiple-comparison procedure was used for the pairwise comparisons of the treatments, using R's multcomp package^(^^29^^)^. Data obtained in the 15 g/d WBE and 15 g/d oligofructose treatment period (i.e. first week of the respective treatment period) were compared with each other and with data obtained in the first week placebo treatment period. Data obtained in the 30 g/d WBE and 30 g/d oligofructose treatment period (i.e. second week of the respective treatment period) were compared with each other and with data obtained in the second week placebo treatment period. For those cases where no significant interactions were found, treatment differences were evaluated based on the main effect model. For those cases where significant interactions were found, treatment differences were evaluated within each treatment sequence group. Next to that, the overall differences were analysed by aggregating over the interaction effects in the model. Aggregation over the interaction effects was done by setting up a linear combination of the treatment differences for each treatment sequence group, giving equal weights to each treatment sequence group. Efficacy results are presented only for the PP population since no differences were observed between the EE and PP results.

### Ordinal measurements from the gastrointestinal symptoms questionnaire

The occurrence frequency and distress severity of all GI symptoms were analysed using the data grouped into ordinal classes. For the analysis of these ordinal data, an ordinal linear mixed-effects model with probit link was used^(^^30^^)^, using the DPolmm function in R's DPpackage^(^^31^^)^. This mixed model covers the correlation of the different measurements within each subject and allows us to model the ordinal data in a similar fashion as the linear mixed model. Treatment × treatment sequence interaction effects were disregarded and only main treatment effects were tested. As this model is a Bayesian model which works iteratively, appropriate starting values were chosen to obtain model convergence. This was tested using Gelman & Rubin's convergence diagnostics. Tests for significant differences between the treatments were done by calculating the 95 % highest posterior density intervals of these treatment differences.

### Statistical analysis of safety variables

The safety population was defined as all randomised subjects who received at least one serving of study product. Safety was analysed using the emergent adverse events (AE) and the changes in clinical blood parameters in the safety population. An AE was attributed to the treatment period during which the AE started. An AE that started during a washout period was attributed to the treatment period preceding the specific washout period. In a first analysis of the changes in clinical blood parameters, the occurrence of adverse shifts in clinical blood parameters (as previously defined by François *et al.*^(^^10^^)^) was determined. McNemar's test was used to compare differences in AE frequencies and differences in blood parameter adverse shifts among the treatments (α = 0·017; Sidak correction for three comparisons^(^^32^^)^). A second analysis of the clinical blood parameters was performed as defined above for the efficacy analysis, but applied to the safety population.

## Results

### Participant characteristics

**Fig. 2.**
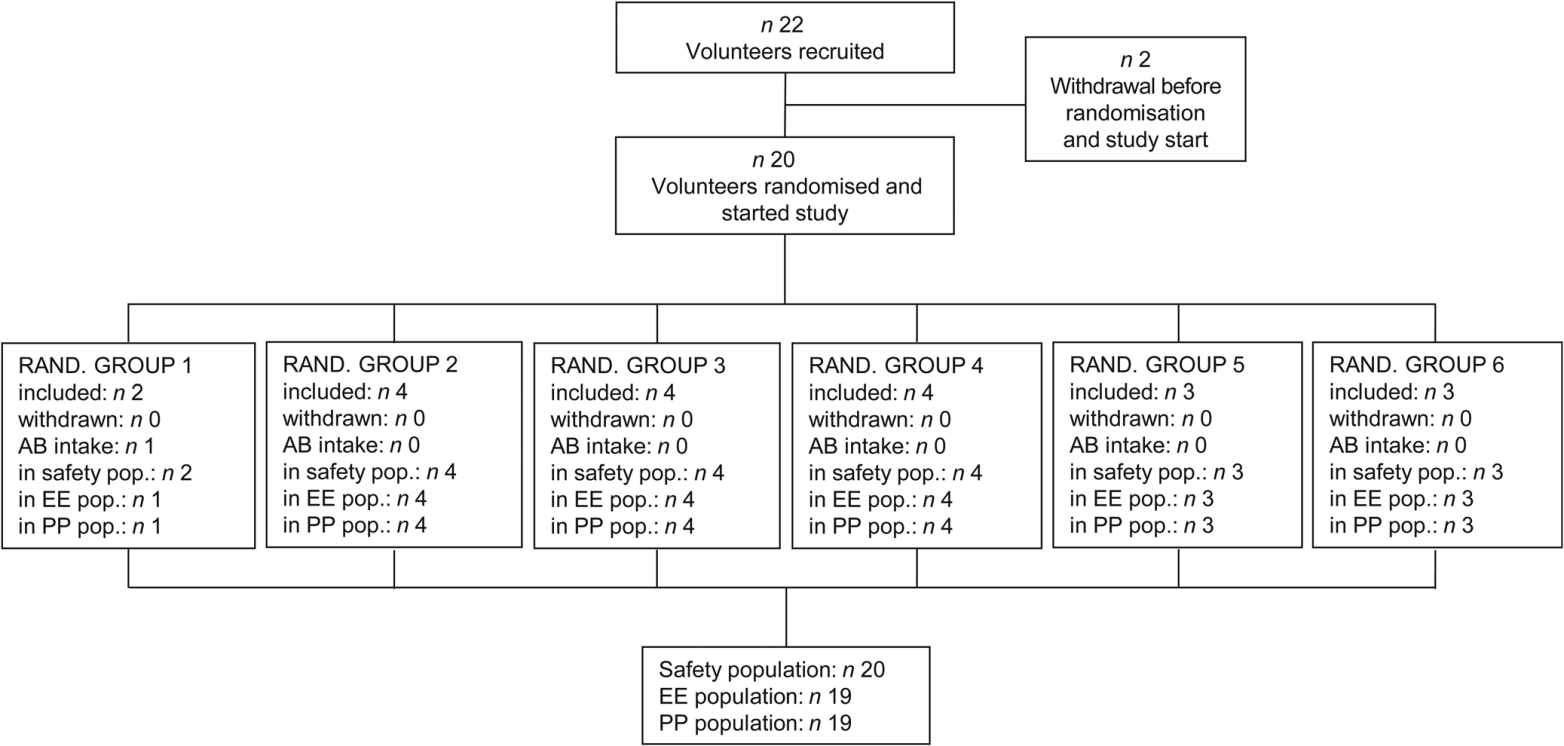
Schematic representation of the volunteer disposition. RAND., randomisation; AB, antibiotics; pop., population; EE, efficacy evaluable; PP, per protocol.

The disposition of all study participants is presented in Fig. 2. A total of twenty-two volunteers were screened and twenty were randomised to any of the six different randomisation groups. None of the volunteers terminated the study prematurely and, hence, twenty volunteers were included in the safety population. One volunteer had to take antibiotics during the course of the study, while all other nineteen volunteers were fully compliant throughout the study. Hence, nineteen volunteers were included in the EE and PP population.

Baseline demographics and anthropometric characteristics for the PP population are presented for the six randomisation groups in Table 4. No significant differences could be observed at baseline between the six randomisation groups with respect to sex, age, BMI and faecal output.

**Table 4.** Baseline characteristics for the six randomisation groups (Number of subjects, and mean values with their standard errors)

		Sex		Age (years)		BMI (kg/m^2^)		Faecal output (g/d)	
Randomisation group	Subjects (*n*)	Female (*n*)	Male (*n*)	Mean	se	Mean	se	Mean	se
1	1	1	0	61·0		29·0		105·4	
2	4	1	3	39·5	7·5	23·8	1·6	99·0	29·4
3	4	3	1	47·5	8·6	21·8	0·6	170·6	13·3
4	4	1	3	47·8	10·7	24·8	1·4	174·7	51·2
5	3	2	1	48·7	12·7	25·7	2·9	196·1	28·0
6	3	1	2	44·0	6·7	24·3	2·6	224·2	119·6
All	19	9	10	46·2	3·7	24·2	0·8	165·4	22·3
Test statistic		0·56*		0·92†		0·46†		0·65†	

* Fisher's exact count data.

† One-way ANOVA.

### Analysis of safety variables

#### Analysis of emergent adverse events

AE were categorised in nine categories according to the National Cancer Institute Common Terminology Criteria for Adverse Events v. 3·0 before unblinding of the study. Statistical analysis of the AE in the safety population revealed no differences between the treatments for any of the nine categories (*P* > 0·1).

#### Analysis of haematological and clinical chemistry parameters

In a first analysis, the occurrence of adverse shifts in forty-four tested clinical blood parameters (listed in Table 3) was determined in the safety population. Statistical analysis indicated no significant differences in occurrence frequency of adverse shifts between the treatments (*P* > 0·1).

A second analysis of the clinical blood parameters was performed as defined for the efficacy analysis, but applied to the safety population (Table 3). Conditional *F* tests showed overall significant treatment effects for only one of the forty-four tested parameters, namely LDL-cholesterol (*P* < 0·1). Subsequent pairwise comparisons demonstrated that intake of 30 g/d oligofructose, but not WBE, modulated LDL-cholesterol levels. The LDL-cholesterol level tended to be higher after the 30 g/d oligofructose treatment (1131·9 mg/l) than after placebo treatment (1024·0 mg/l) (*P* = 0·058). The LDL-cholesterol level after the 30 g/d WBE treatment (1108·0 mg/l) was not significantly different from the LDL-cholesterol level after placebo treatment (*P* > 0·1).

#### Analysis of efficacy variables

Conditional *F* tests showed overall WBE- and/or oligofructose-related significant treatment effects for four parameters (Table 5): stool moisture; stool pH; Bristol Composite Measure; and fibre content of the volunteers' diet (*P* < 0·05). The main results of the subsequent pairwise comparisons of these parameters will be discussed below.

**Table 5.** Efficacy variables following intake of placebo, wheat bran extract (WBE) at 30 g/d or oligofructose at 30 g/d (Mean values and standard deviations)

	Placebo treatment period		30 g/d WBE treatment period		30 g/d oligofructose treatment period		P*			
	Mean	sd	Mean	sd	Mean	sd	*P1*	*P2*	P3	*P4*
Dietary analysis										
Energy intake							0·795	0·786	0·882	0·984
kcal	2385·0	595·7	2300·2	672·0	2313·9	505·3				
kJ	9978·8	2492·4	9624·0	2811·6	9681·4	2114·2				
% Energy from proteins	16·0	4·1	16·0	3·9	15·7	3·7	0·769	1·000	0·809	0·798
% Energy from carbohydrates	52·2	5·7	53·9	6·8	53·3	7·3	0·539	0·501	0·879	0·810
% Energy from lipids	31·8	7·1	30·2	7·8	31·0	7·0	0·463	0·466	0·972	0·621
Fibre content (g)	24·0	12·8	50·8	10·0	51·8	13·3	0·000	0·000	0·000	0·968
Biochemical parameters of faeces										
Stool pH	7·3	0·4	6·8	0·8	6·9	0·8	0·016	0·010	0·060	0·805
Stool moisture concentration (%)	71·4	5·0	74·1	6·2	75·7	5·1	0·003	0·039	0·001	0·451
Faecal output (g/d)	162·3	105·4	183·4	114·4	200·7	122·2	0·232	0·457	0·222	0·879
Bowel habits										
Stool frequency	1·2	0·3	1·1	0·3	1·2	0·4	0·541	0·601	1·000	0·593
Stool consistency	3·8	0·9	4·2	1·2	4·2	1·0	0·201	0·224	0·285	0·989
Bristol composite measure	3·7	0·7	4·2	1·2	4·1	0·9	0·048	0·038	0·135	0·854

* *P1* is the *P* value of the conditional *F* test for overall significant treatment-related effects; *P2*, *P3* and *P4* are the *P* values for the comparison between 30 g/d WBE and placebo, 30 g/d oligofructose and placebo, and 30 g/d WBE and 30 g/d oligofructose, respectively.

### Biochemical parameters of faeces: faecal output, stool moisture concentration and stool pH

Intake of 30 g/d WBE significantly decreased stool pH by about 0·5 units as compared with placebo intake (*P* = 0·001). Intake of 30 g/d oligofructose tended to decrease stool pH by about 0·4 units as compared with placebo intake (*P* = 0·060).

Stool moisture concentration was significantly higher after 30 g/d WBE intake (74·1 %) as compared with stool moisture concentration after placebo intake (71·4 %) (*P* = 0·039). Likewise, stool moisture concentration after 30 g/d oligofructose intake (75·7 %) was also significantly higher than stool moisture concentration after placebo intake (*P* = 0·001).

Faecal output was not modulated by either the 30 g/d WBE treatment or by the 30 g/d oligofructose treatment (*P* > 0·1).

### Bowel habits: Bristol Composite Measure, defecation frequency and stool consistency

The Bristol Composite Measure was significantly higher after the 30 g/d WBE treatment (4·21) as compared with the Bristol Composite Measure after placebo treatment (3·73) (*P* = 0·038). Intake of 30 g/d oligofructose did not modulate the Bristol Composite Measure (*P* > 0·1).

The other bowel habits parameters (defecation frequency and stool consistency) were not modulated by the intake of either 30 g/d WBE or 30 g/d oligofructose (*P* > 0·1).

### Fibre content of diet

The average daily fibre intake of the volunteers during the run-in period was 26·4 (sd 11·3) g/d. Intake of 30 g/d WBE and 30 g/d oligofructose increased the fibre content of the volunteers' diet by about 27 g/d and 28 g/d, respectively, as compared with the fibre content of the volunteers' diet after placebo intake (*P* < 0·001). Neither WBE nor oligofructose intake modulated the average daily energy intake, or the average percentage of energy from carbohydrates, lipids or proteins (*P* > 0·1).

### Tolerability analysis

**Fig. 3.**
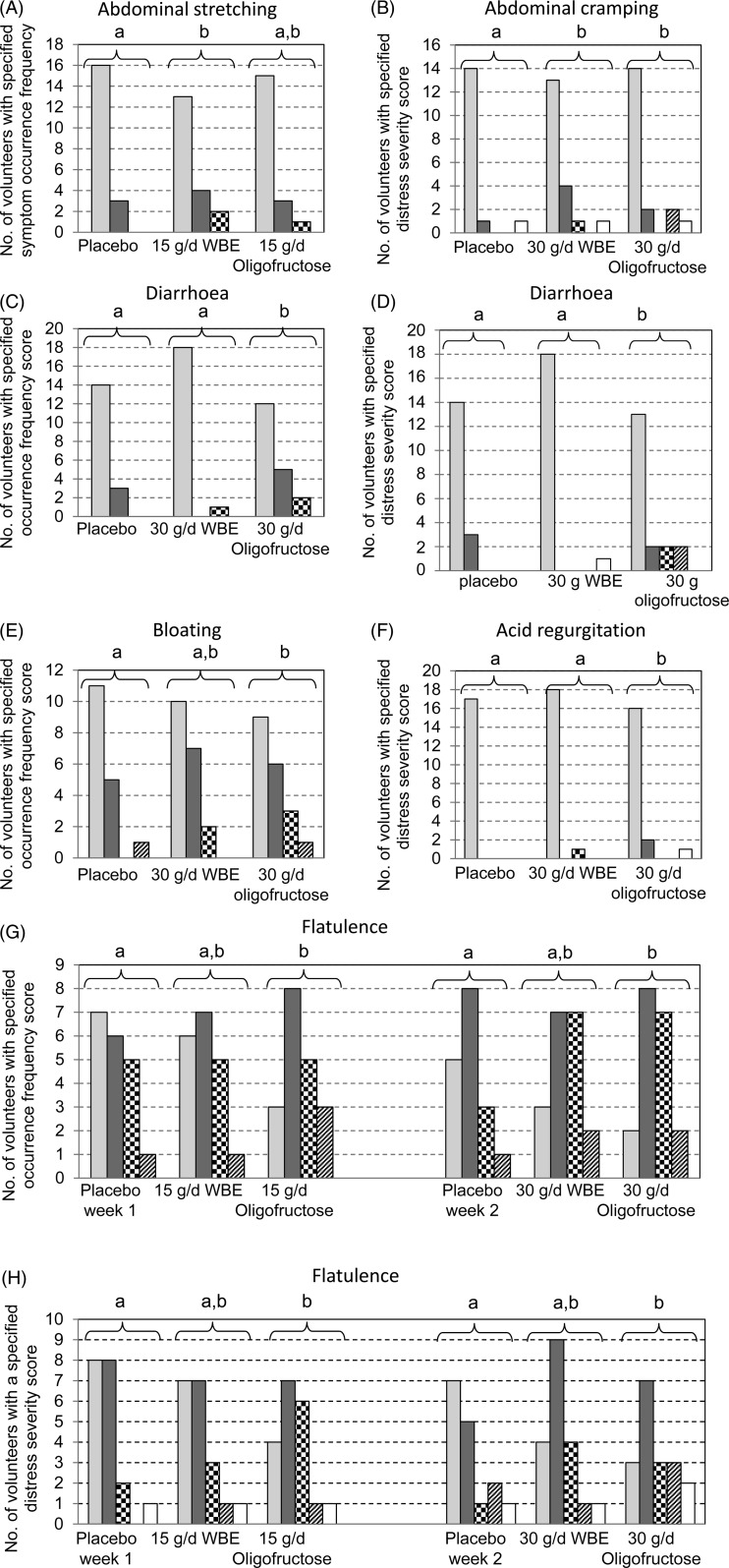
Distribution of occurrence frequency and distress severity of gastrointestinal symptoms. (A) Abdominal stretching occurrence frequency; (B) abdominal cramping distress severity; (C) diarrhoea occurrence frequency; (D) diarrhoea distress severity; (E) bloating occurrence frequency; (F) acid regurgitation distress severity; (G) flatulence occurrence frequency; (H) flatulence distress severity. Scores for occurrence frequency: score 0, never (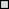); score 1, occasionally (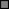); score 2, frequently (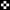); score 3, nearly always (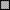). Scores for distress severity: score 0, no distress (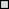); score 1, minimal distress (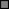); score 2, mild distress (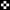); score 3, moderate distress (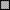); score 4, severe distress (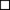). ^a,b^ Groups of bar charts with unlike letters above the brackets represent statistically different distributions (*P* < 0·05). WBE, wheat bran extract.

Tolerability was assessed by self-reported scoring by the volunteers of the occurrence frequency and distress severity of eighteen different GI symptoms. Intake of WBE increased (i) the occurrence frequency of one GI symptom (abdominal stretching) and (ii) the distress severity of one GI symptom (abdominal cramping) as compared with placebo intake (*P* < 0·05). Intake of oligofructose increased (i) the occurrence frequency of three GI symptoms (diarrhoea, flatulence and bloating) and (ii) the distress severity of four GI symptoms (diarrhoea, flatulence, abdominal cramping and acid regurgitation) as compared with placebo intake (*P* < 0·05) (Fig. 3). During treatment with 15 g/d WBE, 2-fold more volunteers experienced abdominal stretching as compared with the first week placebo treatment (Fig. 3(A)). No statistically significant differences in abdominal stretching occurrence frequency were found between the 30 g/d WBE treatment period and the second week placebo treatment period. During treatment with 30 g/d WBE and 30 g/d oligofructose, 3-fold and 2·5-fold more volunteers, respectively, experienced distress from abdominal cramping, as compared with the second week placebo intake (*P* < 0·05) (Fig. 3(B)). During the 30 g/d oligofructose treatment period, 2·3-fold and 7-fold more volunteers experienced episodes of diarrhoea as compared with the second week placebo treatment period and the 30 g/d WBE treatment period, respectively (*P* < 0·05) (Fig. 3(C)). As compared with the second week placebo treatment period and with the 30 g/d WBE treatment period, 2-fold and 6-fold more volunteers experienced distress caused by diarrhoea during the 30 g/d oligofructose treatment period, respectively (*P* < 0·05) (Fig. 3(D)). During the 30 g/d oligofructose treatment period, 1·7-fold more volunteers experienced bloating as compared with the second week placebo treatment (Fig. 3(E)). During the 30 g/d oligofructose intake period, a significant difference in acid regurgitation distress severity was observed between the 30 g/d oligofructose treatment and the second week placebo treatment (*P* < 0·05) (Fig. 3(F)). During the 15 g/d and 30 g/d oligofructose treatment periods, the flatulence occurrence frequency and distress severity were increased as compared with the corresponding placebo treatment period (*P* < 0·05) (Fig. 3(G) and (H)). During intake of oligofructose the number of volunteers that never experienced flatulence was 16 % and 11 % for the 15 g/d and 30 g/d dose, respectively, while it was 37 % and 29 % for the corresponding placebo treatment, respectively. In addition, during intake of oligofructose the number of volunteers that were not experiencing distress due to flatulence was 21 % and 17 % for the 15 g/d and 30 g/d dose, respectively, as compared with 42 % and 44 % for the corresponding placebo treatment. Intake of WBE at doses up to 30 g/d did not modulate the flatulence occurrence frequency in a statistically significant way, nor did it affect the flatulence distress severity as compared with placebo intake (*P* > 0·1).

An overall GI symptom measure was calculated in order to compare the overall effect on GI symptoms across the treatments. Intake of 15 g/d oligofructose caused the overall GI symptom measure (0·36) to increase 2-fold as compared with placebo treatment (0·18) (*P* < 0·05). Intake of 30 g/d oligofructose caused the overall GI symptom measure (0·37) to increase 1·9-fold as compared with placebo treatment (0·20) (*P* < 0·05). Intake of WBE at doses up to 30 g/d did not modulate the overall GI symptom measure (0·26) as compared with placebo intake (0·20) (*P* > 0·1).

## Discussion

WBE and oligofructose intake at a daily dosage of 30 g exerted beneficial effects on stool characteristics. Intake of 30 g/d WBE and 30 g/d oligofructose decreased stool pH by 0·5 units and 0·4 units as compared with placebo intake, respectively. A more acidic colonic lumen is reported to suppress colonisation by pathogens^(^^33^^)^, to reduce the formation of secondary bile acids^(^^34^^)^ and to impair the activity of specific enzymes such as proteases^(^^35^^)^. In addition, a decrease in stool pH could reduce the risk of developing colon cancer, since an inverse correlation between stool pH and colon cancer risk has been observed^(^^36^^)^.

Intake of 30 g/d WBE increased stool moisture concentration (74·1 %) by 2·7 % as compared with placebo intake (71·4 %), whereas intake of 30 g/d oligofructose increased stool moisture concentration (75·7 %) by 4·3 % as compared with placebo intake (71·4 %). The interquartile range (IQR) of stool moisture concentration after placebo intake varied between 64·5 % and 75·0 %. After 30 g/d WBE intake, stool moisture concentration varied between 63·6 % and 78·2 % (IQR). Intake of 30 g/d oligofructose caused stool moisture concentration to vary between 64·4 % and 80·4 % (IQR). Typical stool moisture concentrations range between 70 and 75 %^(^^37^^)^. Stools with a moisture concentration varying between 79 % and 89 % are considered to be diarrhoeal stools^(^^38^^)^. This implies that at least 25 % of the volunteers taking in 30 g/d oligofructose had diarrhoeal stools, which is corroborated by the fact that more volunteers scored a higher diarrhoea occurrence frequency and distress severity upon intake of 30 g/d oligofructose as compared with placebo intake and 30 g/d WBE intake.

Defecation frequency, faecal output and stool consistency were not modulated by intake of either 30 g/d WBE or 30 g/d oligofructose. This was also demonstrated in previous studies using lower dosages of WBE. Intake of 14 g/d WBE by healthy adults did not modulate faecal output^(^^8^^)^. In addition, intake of 10 g/d WBE^(^^10^^)^ and 6·6 g/d WBE^(^^9^^)^ by healthy adults and intake of 5 g/d WBE by healthy children^(^^11^^)^ had no effect on defecation frequency or stool consistency. Likewise, intake of oligofructose was demonstrated not to modulate defecation frequency^(^^39^^)^ or faecal output^(^^15^^,^^39^^,^^40^^)^. In contrast, some studies demonstrate an effect of oligofructose intake on defecation frequency and faecal output^(^^17^^,^^41^^)^, and this upon intake of a lower oligofructose dosage than the dosage used in the present study. In both studies showing an effect of oligofructose intake on faecal output or defecation frequency^(^^17^^,^^41^^)^, the diet was controlled. In the present study, diet was not controlled, which could possibly mask a small effect.

Intake of 30 g/d WBE did not cause adverse shifts in any of the forty-four tested clinical blood parameters, nor did it modulate the values of any of these parameters. Absence of adverse modulations of blood parameters upon intake of WBE at lower dosages was reported in previous trials^(^^8^^,^^10^^)^. While intake of 30 g/d oligofructose also did not induce adverse shifts in any of the forty-four tested clinical blood parameters, it tended to increase LDL-cholesterol levels compared with the placebo treatment (*P* = 0·058). The tendency of oligofructose to increase LDL-cholesterol levels is most probably incidental. Earlier studies on the effect of fructans in human subjects have either shown no significant effect on either blood cholesterol and blood TAG levels^(^^42^^,^^43^^)^, no effect on blood cholesterols and a significant decrease in blood TAG^(^^44^^)^, or a reduction in both blood TAG and blood total cholesterol^(^^45^^)^. In the latter study, daily consumption of 50 g of a rice-based ready-to-eat cereal containing 18 % inulin during 4 weeks was found to significantly (*P* < 0·05) reduce blood total cholesterol and TAG by 7·9 (sd 5·4) % and 21·2 (sd 7·8) %, respectively, compared with the control^(^^45^^)^.

Intake of WBE at doses up to 30 g/d by healthy adults negatively affected only two GI symptoms as compared with placebo intake: abdominal stretching (occurrence frequency) and abdominal cramping (distress severity). Previous studies demonstrated a mild increase in flatulence scores after WBE intake at doses of 10 g/d^(^^10^^)^ and 14 g/d^(^^8^^)^. The other scored GI symptoms were not negatively affected by WBE intake. In the present study, no statistically significant effect on flatulence occurrence frequency or distress severity was found upon intake of 30 g/d WBE. A possible explanation could be that the relatively high basal fibre content of the volunteers' diet in the present study (26·4 g/d) rendered the volunteers tolerant to the mild GI symptom effects caused by WBE.

Intake of oligofructose at doses up to 30 g/d negatively affected five GI symptoms as compared with placebo intake: abdominal cramping (distress severity), diarrhoea (occurrence frequency and distress severity), bloating (occurrence frequency), acid regurgitation (distress severity) and flatulence (occurrence frequency and distress severity). Mild to moderate flatulence has been observed in some studies with oligofructose (at doses ranging between 5 and 20 g/d), which is caused by the production of gases upon fermentation of the prebiotic compound^(^^46^^–^^49^^)^. Stone-Dorshow & Levitt^(^^50^^)^ found that intake of 15 g/d oligofructose during 12 d mildly increased symptoms of abdominal pain, eructation, flatulence and bloating as compared with intake of sucrose as a placebo.

### Conclusion

In conclusion, the data indicate that WBE exerts beneficial effects on stool characteristics, is well tolerated at doses up to 30 g/d and does not cause any adverse effects at up to 30 g/d in healthy adults. Oligofructose exerts comparable beneficial effects on stool characteristics and does not cause any adverse effects at doses up to 30 g/d. However, intake of 30 g/d oligofructose seems to cause GI discomfort to some extent.
